# Linking patient outcome to high throughput protein expression data identifies novel regulators of colorectal adenocarcinoma aggressiveness

**DOI:** 10.12688/f1000research.6388.1

**Published:** 2015-04-24

**Authors:** Christi L. French, Fei Ye, Frank Revetta, Bing Zhang, Robert J. Coffey, M. Kay Washington, Natasha G. Deane, R. Daniel Beauchamp, Alissa M. Weaver

**Affiliations:** 1 Department of Cancer Biology, Vanderbilt University School of Medicine, Nashville, TN, 37232, USA; 2 Department of Biostatistics, Vanderbilt University Medical Center, Nashville, TN, 37232, USA; 3 Center for Quantitative Sciences, Vanderbilt University, Nashville, TN, 37232, USA; 4 Department of Pathology,Microbiology, and Immunology, Vanderbilt University Medical Center, Nashville, TN, 37232, USA; 5 Department of Biomedical Informatics, Vanderbilt University Medical Center, Nashville, TN, 37232, USA; 6 Department of Cell and Developmental Biology, Vanderbilt University School of Medicine, Nashville, TN, 37232, USA; 7 Department of Medicine, Vanderbilt University Medical Center, Nashville, TN, 37232, USA; 8 Department of Veterans Affairs Medical Center, Nashville, TN, 37232, USA; 9 Department of Surgery, Vanderbilt University Medical Center, Nashville, TN, 37232, USA; 10 Vanderbilt Ingram Cancer Center, Nashville, TN, 37232, USA

**Keywords:** Proteomics, Reverse Phase Protein Array, TCGA, Colorectal Cancer, Bioinformatics, Prognosis, Cancer Biology

## Abstract

A key question in cancer systems biology is how to use molecular data to predict the biological behavior of tumors from individual patients. While genomics data have been heavily used, protein signaling data are more directly connected to biological phenotype and might predict cancer phenotypes

such as invasion, metastasis, and patient survival. In this study, we mined publicly available data for colorectal adenocarcinoma from the Cancer Genome Atlas and identified protein expression and signaling changes that are statistically associated with patient outcome. Our analysis identified a number of known and potentially new regulators of colorectal cancer. High levels of insulin growth factor binding protein 2 (IGFBP2) were associated with both recurrence and death, and this was validated by immunohistochemical staining of a tissue microarray for a secondary patient dataset. Interestingly, GATA binding protein 3 (GATA3) was the protein most frequently associated with death in our analysis, and GATA3 expression was significantly decreased in tumor samples from stage I-II deceased patients. Experimental studies using engineered colon cancer cell lines show that exogenous expression of GATA3 decreases three-dimensional colony growth and invasiveness of colon cancer cells but does not affect two-dimensional proliferation. These findings suggest that protein data are useful for biomarker discovery and identify GATA3 as a regulator of colorectal cancer  aggressiveness.

## Abbreviations

CK, Cytokeratin

CRC, Colorectal Cancer

HPA, Human Protein Atlas

IGFBP2, Insulin-like Growth Factor Binding Protein 2

IHC, Immunohistochemistry

RPPA, Reverse Phase Protein Array

TCGA, The Cancer Genome Atlas

TGF-β, Transforming Growth Factor Beta

TMA, Tissue Microarray

## Introduction

High throughput data from the Cancer Genome Atlas (TCGA, https://tcga-data.nci.nih.gov/tcga/) and other publically available datasets are becoming widely available and are a rich resource for data mining and biological discovery. A challenge for the field is to identify innovative approaches to identify both biological drivers and strong prognostic markers. Gene expression datasets have been commonly used to classify tumors, due to their wide availability. However, additional types of high throughput datasets are now available and may provide a different starting point for molecular analysis of tumors. Protein expression datasets generated by mass spectrometry or reverse phase protein array (RPPA) are becoming widely available for many TCGA tumors ^1^. Since gene expression frequently does not correlate well with protein levels ^2^, such datasets may give additional insight into molecular mechanisms that drive tumor behaviors. In addition, phospho-protein levels may identify activation of specific signaling pathways.

A common approach to the analysis of tumor data is to first classify patients by molecular characteristics, such as KRAS mutation status or gene expression clusters, and then determine prognosis or treatment differences ^3– 5^. Alternatively, one can directly identify molecular differences that are statistically associated with patient outcome characteristics. We previously used the latter approach with RPPA data from head and neck squamous cell carcinoma to identify a phosphoinositide 3-kinase high, protein kinase C α low signaling state that drives invasive behavior ^6^. Although it is limited by the availability of patient follow-up data, this type of bioinformatics approach is potentially powerful for identifying novel molecular drivers of tumor aggressiveness.

In this study, we analyzed publicly available data from TCGA to identify proteins that are predictive of poor prognosis in colorectal adenocarcinoma (CRC) ^7^. We analyzed RPPA data, which includes protein and phospho-protein expression levels. Our analysis identified both known and novel candidate CRC drivers statistically associated with tumor recurrence or patient survival. Of these, we characterized two molecules in more detail. IGFBP2 was associated with both death and recurrence. Validation in an independent patient dataset by immunohistochemical (IHC) staining of a tissue microarray (TMA) demonstrated that high levels of IGFBP2 are associated with poor patient prognosis. Interestingly, low protein levels of the transcription factor GATA3 were highly associated with death of CRC patients in the TCGA data set. Experimental studies in colon cancer cell lines indicate that GATA3 expression acts to suppress invasive, aggressive CRC behavior. Since GATA3 protein and RNA levels are not correlated with each other, this association would not have been detected using RNA expression data.

## Experimental procedures

*Antibodies and reagents –* We used three GATA3 antibodies: catalog number 558686 from BD Biosciences (GATA3 BD), catalog number sc-265 from Santa Cruz (GATA3 SC), and catalog number LS-B4163 from LifeSpan Biosciences (GATA3 LS). IGFBP2 antibody was catalog number LS-C138280 from LifeSpan Biosciences and β-actin antibody was catalog number A2228 from Sigma Aldrich. Transwell invasion chambers were from Corning.

*TCGA Data* – RPPA level 3 and clinical information was downloaded from the TCGA data portal. All primary data analyses were performed in R 1.3.1 ^8^.

*Bioinformatics Statistical Analyses* – A univariate Cox’s proportional hazard’s model analysis was performed for each protein (survival package in R) ^9, 10^. Patients with <30 days of follow-up information were excluded. The Wilma algorithm works in a greedy forward strategy and optimizes a combination of the Wilcoxon and Margin statistics for finding clusters of predictor variables (supclust package in R) ^11^. Regsubsets (Leaps package) ^12^ is a model selection method that carries out an exhaustive search for the best subsets of independent variables that predict the dependent variable in linear regression. Nvmax was set to 5 and nbest was set to 10. The RPPA data were median-centered and scaled to one standard deviation before performing analyses. For the Wilma and Regsubsets analyses, patients were divided into good prognosis (living patients or patients with recurrence-free survival were only included if they had ≥ 3 years of follow-up data) or poor prognosis (all patients with a recurrence or death were included regardless of follow-up time).

*Heatmaps* – Heatmaps were created with unsupervised clustering of patients and proteins, using the package “heatmap.plus” in R 1.3.1 based on Euclidian distance and complete linkage ^13^.

*Survival plots* – For each protein, patients were divided into high-expressing (at or above median RPPA expression) and low-expressing (below median RPPA expression). Using SPSS, multivariable cox proportional hazard model was used to estimate overall survival and recurrence-free survival, adjusting for patient stage, and Kaplan-Meier curves were generated to compare survival and recurrence-free survival between high-expressing and low-expressing groups.

*Cell culture*: Cells were grown in previously published optimal media for each cell line (for DLD1 and KM12c, DMEM + 10% FBS and non-essential amino acids) ^14, 15^. DMEM was purchased from Corning, FBS was purchased from Denville Scientific, and non-essential amino acids were purchased from Sigma. To create GATA3-OE cells, DLD1 or KM12c cells were transduced with retrovirus created by transfecting Phoenix packaging cells with pBabePuro-GATA3 (plasmid 1286 from Addgene). Pooled transduced cells were selected by puromycin treatment and used for experiments ^16^. Empty vector pBabePuro was used as a control.

*3D Matrigel growth assay*: Embedded three-dimensional culture was carried out as previously published ^17^. Briefly, 35 mm glass-bottomed Mat-tek dishes (Mat-tek Corporation) were coated with 60 µL Matrigel (Corning). 4,000 cells were plated in each dish in 200 µL 90% Matrigel, 10% growth medium. 2 mL of growth media was added to each dish after 30 minutes and replaced every four days. Cells were imaged at 10× magnification every two days starting at day 3; eight random fields from each dish were imaged and the diameter of each in-focus colony was quantitated.

*Proliferation*: 1500 cells/well were plated in triplicate in the presence or absence of 10% serum in 96 well plates and grown for five days. Each day the plates were imaged on a Cellavista automated microscope after the addition of Calcein to identify live cells, Propidium iodide to identify dead cells, and Hoechst to identify nuclei (all from Invitrogen). Data were quantitated with Cellavista imaging software to determine the number of live cells for each day.

*Transwell invasion assay*: 50,000 cells/well were plated in triplicate on Matrigel-coated Transwell inserts in serum-free DMEM. Normal growth media was used on the bottom as a chemoattractant. Cells were allowed to invade for 48 hours and then fixed with a three-step stain (Thermo Scientific). Five random fields from each Transwell insert at 10× magnification were taken on an EVOS microscope for quantitation.

*Tissue microarray construction and IRB information*: All use of human tissue samples was conducted under IRB-approved protocols. The colorectal cancer tissue microarray (TMA) was constructed with 99 cases of colorectal cancer, using duplicate 1-mm cores of each colorectal cancer in the GI SPORE Tissue Core facility (IRB # 020338). All samples in the TMA are from formalin-fixed paraffin-embedded blocks in the pathology archives, and are from tissue removed during the course of routine clinical care. Associated outcome and demographic data are extracted from the Colorectal Carcinoma Data and Virtual Archival Specimen Repository (IRB# 101531), and are stripped of all identifiers when released to investigators. The array is enriched for special histologic subtypes of CRC such as mucinous, signet ring cell, and medullary carcinoma, and contains the full spectrum of histologic grades and tumor stages. Twelve control cases of histologically normal colorectal mucosa from surgical resections for non-neoplastic disease such as diverticulosis coli are included.

*TMA staining*: Antigen retrieval was performed in pH 6.0 citrate buffer, by using a pressure cooker at 104°C for 20 minutes with a 10 minute bench cool down, followed by quenching with 0.04% H _2_O _2_ w/sodium azide for 5 minutes. After blocking in a serum-free protein block for 20 min, primary antibody was incubated with the samples for an hour, followed by detection with Dako Envision + HRP Labeled Polymer for 20 minutes followed by incubation with chromogen DAB+ for 5 minutes.

*TMA analysis*: To be included in the survival or recurrence-free curves, patients needed to have the following information: stage, days until event (if deceased or recurrent), and a follow-up time of at least 30 days (if living or nonrecurrent). Through the Vanderbilt University Digital Histology Shared Resource in the Epithelial Biology Center, immunostained TMA slides were imaged at 20× magnification to a resolution of 0.5 µm/pixel with the Leica SCN400 Slide Scanner (Leica Biosystems). Tissue cores were analyzed with Ariol® Review software SL-50. Upper and lower thresholds for brown DAB positive staining were set for color, saturation, and intensity. Tumor areas with staining that registered between these thresholds were determined to be DAB-positive in an automated analysis. Brown (DAB-positive) area of each tumor core was thus used to determine cytokeratin (tumor area), IGFBP2, and GATA3 stained area. The percent of the tumor area positive for IGFBP2 was calculated by dividing the IGFBP2- positive area by the cytokeratin-positive area and multiplying by 100.

*Numbers and statistics*: For comparison of good and poor prognosis patients, a Fisher’s exact test was used to analyze categories with two variables (gender, M). A Chi-squared test was used to analyze categories with more than two variables (Stage, T, N). Age and gender were analyzed using a Student *t*-test. All analyses were performed in GraphPad. For experimental data from CRC cell lines, data from the engineered cell lines were plotted and statistically analyzed in GraphPad using a Student *t*-test. Data plotted in bar graphs were represented as mean+/-standard error. For growth curves, error bars represent 95% confidence intervals.

## Results

To identify molecular drivers of aggressive CRC behavior, we used statistical methods to link patient outcome data to protein and phospho-protein expression in the TCGA RPPA dataset. The RPPA dataset includes protein and phospho-protein levels from tumor biopsies taken at the time of diagnosis. The clinical information for these patients is also available, including recurrence and survival information, stage, and follow up time ( Table 1, Table 2; Datafile 1).

**Table 1.** Characteristics of patients with RPPA data included in Wilma and Regsubsets analyses for death and recurrence.

*Patients included in Wilma and Regsubsets analyses*											
	*Recurrence*			*Death*		
	Recurrent	Non-recurrent (3 yr. follow-up)	p-value	Deceased	Living (3 yr. follow-up)	p-value
*Total number*	22	12		23	20	
*Average age*	66.86	59.83	0.2339	73.96	63.65	0.0274*
*Average weight*	77.55	79.75	0.6605	67.21	77.79	0.1732
*Male*	13	7	1	14	10	0.5472
*Female*	9	5		9	10	
*Stage I*	0	2	0.0341*	3	3	0.2379
*Stage II*	6	2		6	5	
*Stage III*	6	7		4	8	
*Stage IV*	9	1		9	3	
*T0*	0	0	0.0834	1	0	0.1315
*T1*	0	1		0	1	
*T2*	0	2		4	3	
*T3*	17	9		11	15	
*T4*	5	0		7	1	
*N0*	7	4	0.3392	10	8	0.9904
*N1*	8	7		8	7	
*N2*	6	1		5	4	
*M0*	7	10	0.0161*	10	13	0.1516
*M1*	10	1		9	3	

**Table 2.** Characteristics of patients with RPPA data included in Cox regression analysis for death and recurrence.

*Patients included in Cox regression analysis*											
	*Recurrence*			*Death*		
	Recurrent	Non-recurrent (3 yr. follow-up)	p-value	Deceased	Living (3 yr. follow-up)	p-value
*Total number*	22	125		23	168	
*Average age*	66.86	63.53	0.3418	73.96	65.21	0.003*
*Average weight*	77.55	82.84	0.2136	67.21	82.62	0.0459*
*Male*	13	63	0.4951	14	86	0.5052
*Female*	9	62		9	82	
*Stage I*	0	22	0.0009*	3	27	0.0016*
*Stage II*	6	46		6	67	
*Stage III*	6	41		4	53	
*Stage IV*	9	13		9	17	
*T0*	0	0	0.1259	1	0	0.0037*
*T1*	0	3		0	3	
*T2*	0	22		4	27	
*T3*	17	87		11	120	
*T4*	5	10		7	15	
*Tis*	0	1		0	1	
*N0*	7	75	0.0137*	10	102	0.2263
*N1*	8	39		8	45	
*N2*	6	11		5	20	
*M0*	7	99	< 0.0001*	10	130	0.0006*
*M1*	10	13		9	18	

Therefore, we used a combination of univariate and multivariate approaches to identify proteins associated with recurrence or death. Univariate Cox proportional hazard regression analysis ^9, 10^ relates the time to an event to a covariate (gene or protein expression) and is a common method to identify associations of protein expression with patient outcome. We also used Wilma and Regsubsets multivariate algorithms to select groups of proteins with predictive power ^12, 18^. Patient characteristics are shown in Table 1 for the Cox regression analysis and in Table 2 for the Wilma/Regsubsets analyses. The use of all 3 methods allowed us to identify whether certain proteins were chosen independent of the statistical method used.

**Supplemental Figure1.**
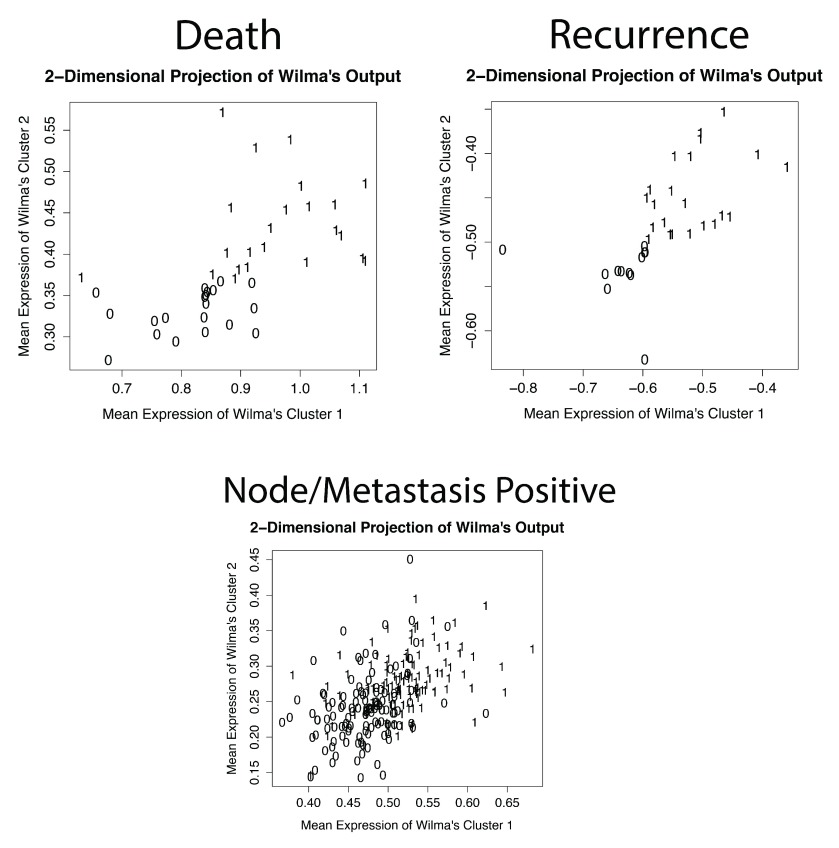
Comparison of death, recurrence, and node/metastasis status as metrics to identify proteins with the ability to predict patient prognosis. All number indicate individual patients; 1’s are patients with poor prognosis (death; recurrence; N or M positive at time of diagnosis) and 0’s are patients with good prognosis (living with 3 years of follow up time; non-recurrent with 3 years of follow up time; N and M negative at time of diagnosis). The distinct populations in the death and recurrence plots, showing clear separation of the good and poor prognosis patient clusters, indicate these definitions of poor prognosis can identify groups of proteins with good predictive power. The overlap of these patient clusters in the Node/Metastasis plot indicates this definition has less predictive power.

The Wilma and Regsubsets algorithms compare groups (clusters) of patients, which we predefined by patient prognosis, and find proteins that are able to predict these clusters. For these multivariate methods, patients were divided into “good” or “poor” prognosis groups according to survival or recurrence data. “Good prognosis” patients were classified either as living or as having no recurrence with a minimum of 3 years follow-up time. We chose 3 years as a reasonable cut-off time since the great majority of colon cancer cases (91%) have a recurrence within this time frame ^19^. Although this did reduce our sample size for patients included in the multivariate analyses compared to the univariate Cox regression ( Table 1 vs. Table 2), we felt it was necessary to ensure that our “good prognosis” group was accurate. For the “poor prognosis” patient group, recurrence or death could occur at any time point. To determine whether any proteins had stage-specific statistical associations, we performed the analyses using patient groups of stages I-II, stages I-III, or stages I-IV ("all stages"). However, we did not use stage, node or metastasis status as traits for identification of molecular correlates for several reasons. First, we reasoned that identifying molecular correlates of stage would not add prognostic information for clinical decision making, since stage is already gathered on every patient. Second, an initial test using the Wilma algorithm suggested that RPPA protein expression changes selected to be associated with node and metastasis negativity (e.g. N0M0 vs. N+M+) did not segregate patients well into groups. Thus, two-dimensional projections indicate that proteins selected by both recurrence and death had the ability to separate patients into distinct groups, indicating good predictive power, while N/M status at the time of diagnosis did not ( Supplemental Figure 1).

**Figure 1.**
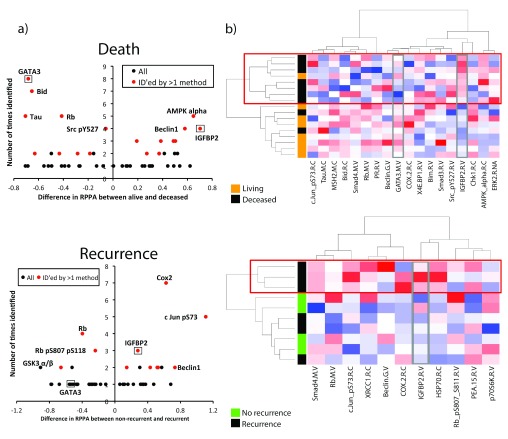
Visualization of proteins identified by bioinformatics analysis. **a**) Volcano plots were created by plotting the difference in the scaled RPPA expression for each protein vs. the number of times that protein was identified in the bioinformatics analysis. A positive value on the y-axis means that protein is upregulated in poor prognosis (recurrent or deceased) patients, while negative value on the y-axis means that protein is downregulated in poor prognosis (recurrent or deceased) patients. Proteins identified by more than one bioinformatics method ( Table 3, Table 4) are shown in red, and proteins selected for further analysis are boxed and labeled. **b**) Heatmaps were created using unsupervised clustering of all top hits ( Table 3, Table 4) in stage I-II patients. Each row is a patient; each column is a protein. Red boxes outline poor prognosis (recurrence or death) clusters. Proteins selected for further analysis (GATA3 and IGFBP2) are outlined in grey boxes.

The full results of the analyses for molecules statistically associated with death or recurrence are shown in Supplemental Table 1– Supplemental Table 4 (Cox hazard analyses shown in Supplemental Table 1, Supplemental Table 2, and results from all analyses summarized in Supplemental Table 3, Supplemental Table 4). Modified volcano plots of these proteins shows the number of times a protein was identified vs. the difference in RPPA expression for either death or recurrence ( Figure 1a). Proteins with negative values are downregulated in patients with poor outcome (such as the well- known tumor suppressor, Rb) and proteins with positive values are upregulated (such as the oncogene c-Jun). Proteins that were identified by more than one method are shown in Table 3 and Table 4 and indicated in red in the volcano plots ( Figure 1a).

**Table 3.** Summary tables for death, ordered by the number of times each protein was selected. Proteins that were identified by more than one computational method (Cox regression, Wilma, or Regsubsets) were included. Proteins identified by Cox regression and the Wilma algorithm were significantly associated with prognosis (p<0.05); proteins are included for Regsubsets if they were identified five times or more.

*Death*											
	Method	Cox			Wilma			Regsubsets			
	*Stages*	*All*	*I-II*	*I-III*	*All*	*I-II*	*I-III*	*All*	*I-II*	*I-III*	Total #
**Antibody**	GATA3.M.V	✔	✔	✔	✔	✔	✔		✔	✔	8
	Bid.R.C	✔		✔		✔	✔	✔	✔	✔	7
	Rb.M.V		✔	✔		✔	✔			✔	5
	AMPK_alpha.R.C	✔	✔	✔	✔	✔					5
	Tau.M.C	✔	✔	✔	✔			✔			5
	IGFBP2.R.V	✔	✔	✔			✔				4
	Beclin.G.V				✔		✔	✔		✔	4
	Src_pY527.R.V				✔	✔			✔	✔	4
	COX.2.R.C				✔			✔		✔	3
	c.Jun_pS73.R.C					✔	✔			✔	3
	X4E.BP1.R.V					✔	✔		✔		3
	Bim.R.V	✔			✔			✔			3
	Smad4.M.V				✔		✔				2
	ERK2.R.NA				✔				✔		2
	PR.R.V		✔	✔							2
	Chk1.R.C					✔		✔			2
	MSH2.M.C				✔				✔		2
	Smad3.R.V	✔				✔					2

**Table 4.** Summary tables for recurrence, ordered by the number of times each protein was selected. Proteins that were identified by more than one computational method (Cox regression, Wilma, or Regsubsets) were included. Proteins identified by Cox regression and the Wilma algorithm were significantly associated with prognosis (p<0.05); proteins are included for Regsubsets if they were identified five times or more.

*Recurrence*											
	Method	Cox			Wilma			Regsubsets			
	*Stages*	*All*	*I-II*	*I-III*	*All*	*I-II*	*I-III*	*All*	*I-II*	*I-III*	Total #
**Antibody**	COX.2.R.C	✔	✔	✔	✔		✔	✔	✔		7
	c.Jun_pS73.R.C	✔			✔		✔	✔		✔	5
	Rb.M.V					✔	✔		✔	✔	4
	IGFBP2.R.V		✔	✔			✔				3
	Rb_pS807_S811.R.V					✔		✔	✔		3
	Beclin.G.V				✔		✔				2
	Smad4.M.V						✔			✔	2
	HSP70.R.C		✔		✔						2
	p70S6K.R.V				✔			✔			2
	PEA.15.R.V		✔						✔		2
	XRCC1.R.C			✔			✔				2

Proteins associated with death included known CRC drivers, including SMAD3, SMAD4, and MSH2, which respectively regulate Transforming growth factor beta (TGF-β) signaling ^20^ and microsatellite instability ^21^ ( Table 3). In addition, a number of apoptosis and cell cycle proteins were associated with death, including Bid, Bim, Rb, and Chk1. Interestingly, the transcription factor GATA3 was our top hit associated with patient death and was identified eight times out of a potential maximum of nine times (three stage groups analyzed by three statistical methods). GATA3 is frequently mutated in breast cancer and is known to promote luminal cell differentiation in the mammary gland ^22– 25^, but has not been previously studied in colon cancer. IGFBP2, which was linked with both patient death and tumor recurrence in our analysis, was another interesting hit, as it has been associated with a number of cancer types but few studies have addressed its role in CRC ^26– 28^.

Proteins associated with recurrence ( Table 4) also included known CRC regulators, including the pro-inflammatory enzyme COX2 ^29, 30^, phospho-c-Jun ^31^ and SMAD4 (reviewed in 32). Some proteins were identified to be statistically associated with both death and recurrence, including the cell cycle regulator Rb, the autophagy regulator Beclin1, and IGFBP2.

To visualize the expression of top hits (listed in Table 3 and Table 4) in individual patient tumor samples, we created heatmaps using unsupervised clustering. Interestingly, clustering of data from Stage I and II patient tumors gave superior segregation of prognosis groups by the proteins than using data from Stages I-III or I-IV patient tumors. For both recurrence and survival, there was a “poor prognosis” cluster that segregated away from the remaining patients ( Figure 1b, red boxes). Notably, the ability of the chosen proteins to cluster patients according to poor prognosis was also superior when using death as the outcome, perhaps due to the larger number of significant proteins or the larger sample size of Stage I-II patients with that follow-up metric ( Figure 1b, compare death and recurrence heat maps).

**Figure 2.**
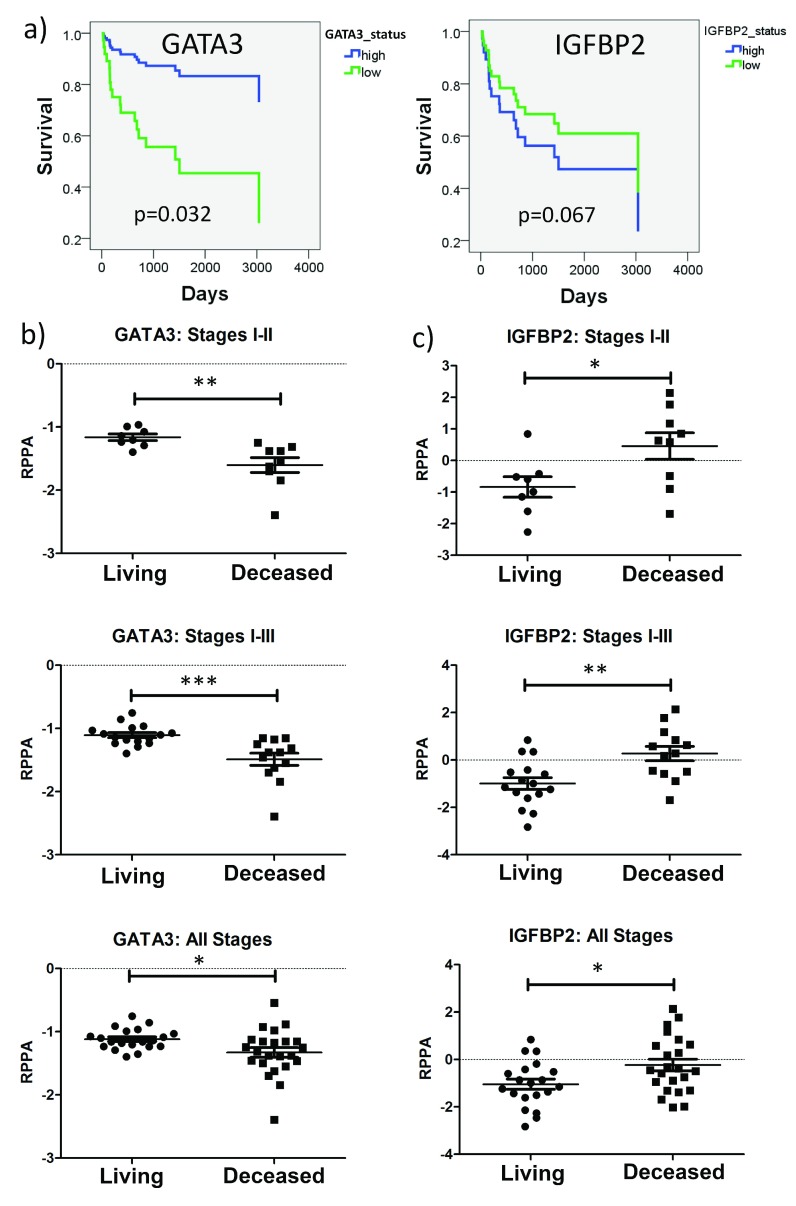
Survival analysis of selected proteins in TCGA data. ( **a**) Stage-adjusted survival plots for GATA3 and IGFBP2. ( **b**) and ( **c**) Comparison of RPPA-determined expression in living and deceased patients for GATA3 ( **b**) and IGFBP2 ( **c**). IGFBP2 expression is significantly increased in deceased patients in Stages I-II, I-III, and I-IV, while GATA3 is significantly decreased in deceased patients in Stages I-II, I-III, and I-IV. *p<0.05, **p<0.01, ***p<0.001

**Supplemental Figure 2.**
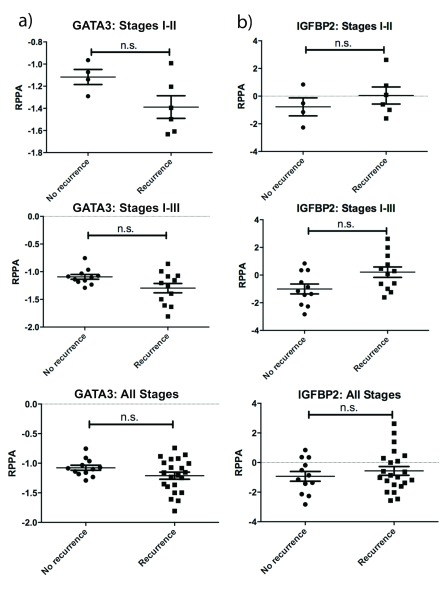
Comparison of RPPA-determined expression in non-recurrent and recurrent patient tumors for GATA3 and IGFBP2. Decreased GATA3 ( **a**) and increased IGFBP2 ( **b**) expression are evident in recurrent patient tumors, but the data were not significant (n.s.).

Of the proteins identified in our analyses, GATA3 and IGFBP2 were the most novel as regulators of CRC. Visualization by heatmaps shows a decreased expression in GATA3 and increased IGFBP2 expression in tumors within the poor prognosis clusters ( Figure 1b, grey boxes). Stage-adjusted survival plots revealed that TCGA patients with low GATA3 expression levels had a significantly increased risk of death, compared with patients whose tumors had high GATA3 levels. Patients whose tumors had high IGFBP2 expression had a trend towards decreased survival, but this did not reach statistical significance ( Figure 2a). Importantly, both GATA3 and IGFBP2 had significantly altered RPPA expression in deceased patients for all stages, stages I-II, and stages I-III ( Figure 2b, c). Similar trends were seen in recurrent vs. non-recurrent patients, but the data did not reach statistical significance, potentially due to the smaller number of patients with recurrence follow up data ( Supplemental Figure 2).

**Figure 3.**
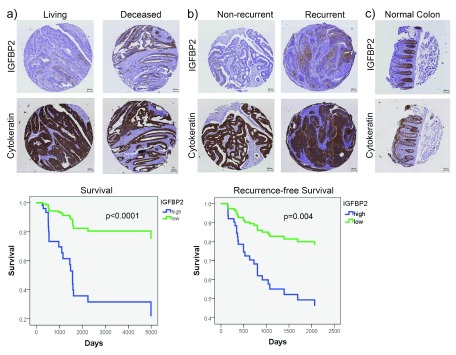
IGFBP2 expression is associated with recurrence and death in CRC in a secondary dataset. IHC immunostaining of a CRC tissue microarray for IGFBP2 and cytokeratin (epithelial marker) was performed. **a**) Representative IGFBP2 staining in living and deceased patients and Kaplan-Meier curve comparing survival of patients with low (below median) vs. high (at or above median) IGFBP2 staining. **b**) Representative IGFBP2 staining in non-recurrent and recurrent patients and Kaplan-Meier curve comparing recurrence-free survival of patients with low (below median) vs. high (at or above median) IGFBP2 staining. %IGFBP2-positive area of tumor was calculated using IGFBP2 area and cytokeratin area to identify tumor. Survival and recurrence-free survival plots are adjusted for stage. **c**) Representative IGFBP2 and cytokeratin staining in a representative normal colon sample. Scale bars indicate 100 µm.

To validate our findings in an independent tumor cohort, we obtained a tissue microarray (TMA) that contained 61 CRC samples with available patient follow-up data ( Datafile 2). Patient characteristics are shown in Supplemental Table 5. Note that some clinical information, such as age or gender, was not available for all patients. We stained the TMA slides with antibodies against IGFBP2 as well as with the epithelial marker cytokeratin in order to identify tumor cells ( Figure 3a, b). We quantified the areas of both IGFBP2 staining and cytokeratin staining (representing total tumor area), and calculated the percent IGFBP2 positive area per tumor area in order to normalize to the amount of tumor present in each sample ( Datafile 2). This metric was used to divide patients into high or low IGFBP2 by median expression, and their survival or recurrence-free survival was compared. The results revealed that patients with IGFBP2 staining at or above the median had a significant reduction in both survival and recurrence-free survival time, independent of tumor stage ( Figure 3a, b, lower panels). Staining of normal colon tissue also revealed strong staining in the bottom of the crypts ( Figure 3c), consistent with a previous report ^28^.

**Figure 4.**
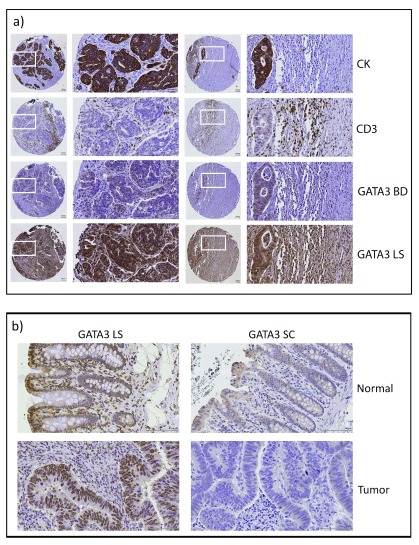
GATA3 is expressed in human CRC. **a**) Representative immunostained tissue sections from two patient tumors from the CRC TMA showing staining for epithelial tumor (cytokeratin, CK), T-cells (CD3), and two different GATA3 antibodies (BD and LS). **b**) Representative staining of matched normal colonic tissue and colon cancer samples for two different GATA3 antibodies (LS and SC). Note the variability of GATA3 staining with different antibodies.

GATA3 is a transcription factor that was originally identified as a T-cell differentiation factor ^33, 34^. However, recent data indicates that GATA3 is also expressed in some epithelia (reviewed in 35). In breast cancer, GATA3 is frequently mutated ^23, 25^. In addition, low levels of GATA3 correlate with decreased breast cancer patient survival ^36– 40^. To determine whether GATA3 was expressed in CRC cells or only in T-cells, we stained CRC TMAs as well as matched normal and colon cancer tissue ( Figure 4; Datafile 3). Antibodies to cytokeratin (CK) and CD3 respectively marked the epithelial tumor cell and T-cell compartments. We found variable staining patterns with two different anti-GATA3 antibodies. Using the same antibody that was used to probe the TCGA RPPA samples ( Figure 4a , GATA3 BD), there was weak cytoplasmic and occasional nuclear staining in the tumor cells and a small amount of nuclear staining in cells in the stromal compartment. It should be noted that this antibody had not been validated for IHC. Furthermore, we noticed variable staining of TMA sections from normal colon tissue, suggesting high sensitivity of this antibody to fixation conditions. We therefore tested two more antibodies that were validated for IHC. Using an antibody that has successfully been used for breast cancer stratification ^36^, we detected very light cytoplasmic staining of epithelial cells with some nuclear staining of stromal cells in normal colon samples, but no staining of epithelial or stromal cells in paired colon cancer samples (GATA3 SC, Figure 4b). Using a second validated IHC antibody (GATA3 LS), we found strong staining of the epithelial component of both normal colon tissue and colon cancer ( Figure 4b). Interestingly, with both the SC and LS antibodies, it appeared that in normal colon tissue there was increased staining in epithelial cells at the mucosal surface with nuclear localization, compared to the deep crypts ( Figure 4b). Staining of the TMA with GATA3 LS gave strong staining in both the nuclei and cytoplasm of tumor cells. However, there was a high background in many of the samples with apparently nonspecific staining throughout both the tumor and stromal compartment ( Figure 4a), which made the samples unsuitable for quantitation. This high background may be due to overfixation of some of the TMA blocks, since it was not apparent on separate fixed tissues that were not part of the TMA (compare Figure 4a to Figure 4b , GATA3 LS staining).

**Supplemental Figure 3.**
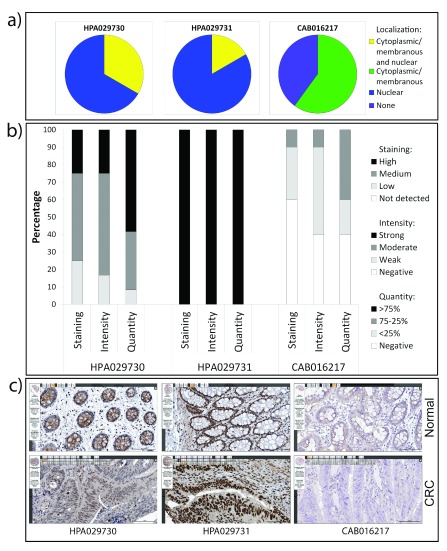
Comparison of GATA3 staining patterns in CRC tumor samples in the Human Protein Atlas (HPA) using three different antibodies. **a**) Subcellular localization; **b**) Staining, intensity, and quantity plots from the HPA. **c**) Representative images from matched normal colon tissue and CRC samples with three different GATA3 antibodies, as indicated.

We also checked the Human Protein Atlas (HPA) ^41^ for staining of colon tissues by GATA3 antibodies ( Supplemental Figure 3). The HPA also used three different antibodies. One of them, CAB016217, is the same as the antibody we tested that gave little to no staining of colon tissue (GATA3 SC). Likewise, they found little nuclear staining, and weak or negative cytoplasmic staining across both normal and colon cancer samples. The other two antibodies stained the epithelial component of both normal and colon cancer samples with primarily nuclear or nuclear + cytoplasmic staining patterns. Thus, with four out of the five antibodies tested by our laboratory and the HPA, nuclear GATA3 staining was seen in colon epithelial and cancer cells. However, due to the variability in intensity and pattern of staining, we were not able to perform quantitations to obtain information about prognostic significance.

**Supplemental Figure 4.**
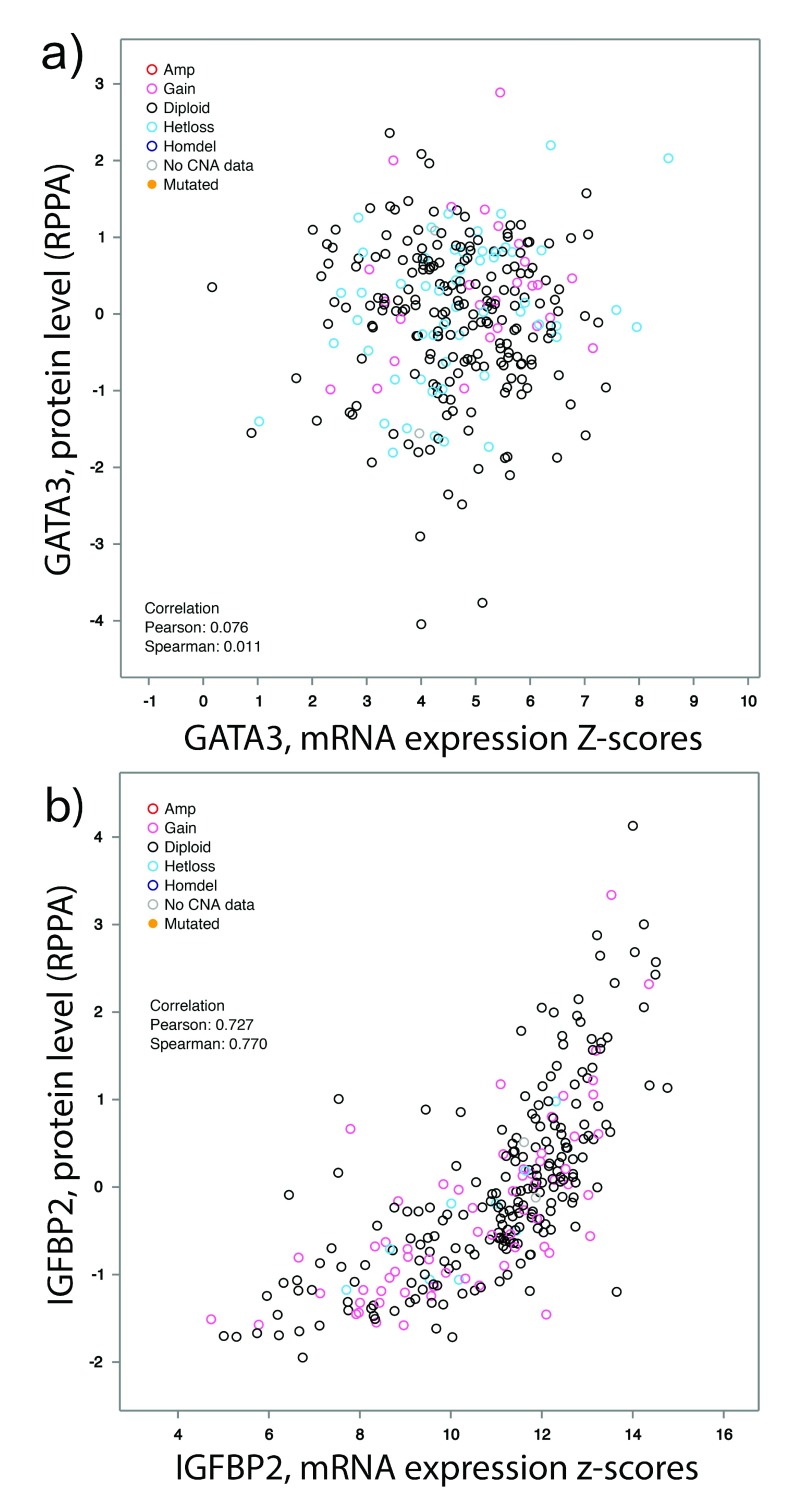
Correlations of RPPA protein expression with RNA expression. GATA3 and IGFBP2 RPPA and mRNA expression values from TCGA datasets were plotted and analyzed on an individual tumor basis. ( **a**) GATA3 mRNA expression does not correlate with protein expression. ( **b**) IGFBP2 mRNA expression does correlate with protein expression. Plots were created with cBioPortal using TCGA (2012) dataset ^6^.

To determine if we could use a gene expression dataset for validation, we tested whether GATA3 RNA expression by RNA sequencing correlated with GATA3 protein expression by RPPA in TCGA samples that had both types of data. There was no correlation between GATA3 RNA and protein expression ( Supplemental Figure 4a), so we were not able to use GATA3 RNA expression for correlative studies in a secondary tumor dataset. By contrast, IGFBP2 protein levels correlate well with IGFBP2 RNA levels ( Supplemental Figure 4b). There was no correlation between IGFBP2 protein and GATA3 protein levels (data not shown), indicating there is likely no mechanistic link between these two proteins.

**Figure 5.**
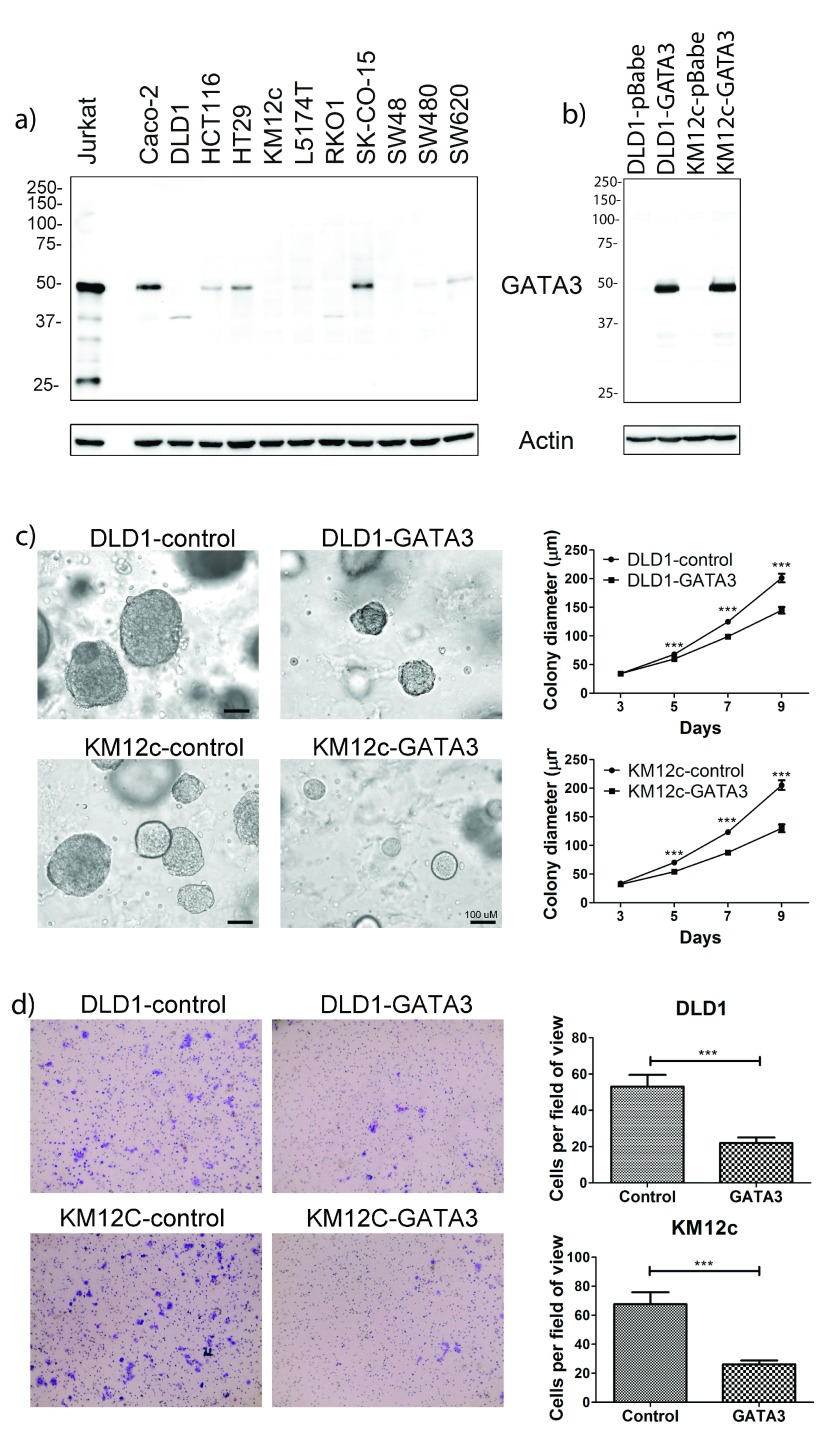
GATA3 expression affects CRC aggressiveness. **a**) Representative Western blot (of 2 blots) showing that GATA3 is expressed in a subset of CRC cell lines. Jurkat is a T-cell line and used as a positive control. Higher expression is seen in the more differentiated cell lines Caco-2, HT-29, and SK-CO-15. **b**) Western blot showing engineered expression of GATA3 in DLD1 and KM12c CRC cell lines. pBabe is an empty vector control. **c**) Colony growth of engineered CRC cell lines in 3D Matrigel. Left: Representative images from day 9. Right: Growth curves. Data were gathered from duplicate wells from 3 independent experiments. The mean is plotted and error bars represent 95% CI. **d**) Invasion of CRC cell lines across Transwell filters. Left: Representative images of the bottom of Transwell filters after 48 hours invasion. Right: Quantitation of invaded cells/field. Data from five random fields per filter x triplicate filters for each of 3 independent experiments. Error bars represent +/- SEM. ***p<0.001.

As an alternative to validation with tissue samples, we decided to investigate the biological role of GATA3 in colon cancer with *in vitro* experiments. We performed Western blot analysis of GATA3 levels in a panel of CRC cell lines with Jurkat T-cells as a positive control for GATA3 expression ( Datafile 4). Using the same antibody that was used in the TCGA RPPA analyses (GATA3 BD), we detected a band of the correct 48 kDa size for GATA3. Compared with Jurkat cell expression, GATA3 was expressed at a much lower level in most CRC cell lines. GATA3 expression was undetectable in about half of the cell lines tested, including several with invasive characteristics, e.g. DLD1, SW480, and SW620 ^42, 43^. Consistent with the known role of GATA3 in cellular differentiation ^34, 44– 48^, the highest GATA3 expression was observed in the more differentiated cell lines, Caco-2, SK-CO-15 and HT-29 ^49– 51^ ( Figure 5a).

**Supplemental Figure 5.**
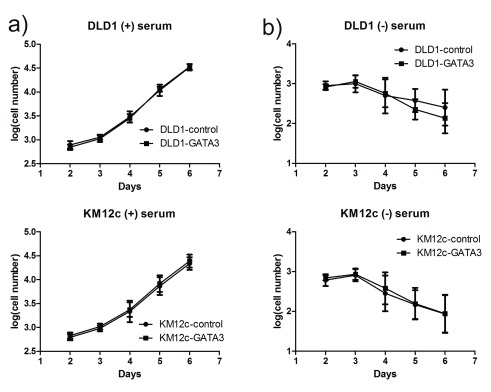
GATA3 does not affect 2D CRC proliferation. Growth curves (log base 10 of the cell number) from CRC cell lines grown in the presence of 10% serum ( **a,** "(+) serum)") or the absence of serum ( **b,** "(-) serum"). Cells were plated in triplicate and imaged on a Cellavista automated microscope in 3 independent experiments. Mean is plotted and error bars represent 95% confidence intervals. No significant differences were observed between control and GATA3-OE cells for either cell line.

To investigate the role of GATA3 in CRC growth and invasion, we chose two of the invasive cell lines with undetectable GATA3 expression and stably expressed GATA3 in them using retroviral transduction ( Figure 5b; Datafile 4). We first tested the ability of the GATA3-expressing cells to form colonies after seeding as single cells in an embedded 3D Matrigel growth assay. Colony growth in this assay represents a combination of growth and matrix remodeling activity, since the cells are fully embedded in 90% Matrigel ^52– 54^. Compared with control cells, GATA3-expressing cells formed smaller colonies in this 3D culture environment, an effect that was statistically significant beginning at day 5 ( Figure 5c; Datafile 5). To determine whether the smaller colony size of GATA3-expressing cells was due to an intrinsic decrease in proliferation rate, we cultured them in 2D in the presence or absence of serum and used automated microscopy to follow the number of cells over a period of 5 days. GATA3 expression had no effect on cell numbers in the presence or absence of serum ( Supplemental Figure 5; Datafile 6). To determine if GATA3 specifically controls CRC invasiveness, control and GATA3-expressing cells were allowed to invade for 48 h across a bed of Matrigel in a Transwell invasion assay. For both of the tested CRC cell lines, GATA3-expressing cells exhibited significantly decreased invasion compared to control cells ( Figure 5d; Datafile 7). Taken together, these data indicate that GATA3 controls CRC invasiveness.

Raw data of identified protein expression and signaling changes statistically associated with patient outcome Detailed legends describing the each data files are can be found in the .txt file provided.

## Discussion

In this study, we used high throughput protein and phospho-protein expression data from the TCGA to identify candidate drivers of CRC aggressiveness. By linking RPPA data to patient death or recurrence and using multiple statistical approaches, we identified both known and novel biomarkers of CRC aggressiveness. The top hit in our survival analysis was the transcription factor GATA3, for which low levels correlated with death. Follow-up experiments indicated that GATA3 is expressed in CRC and suppresses the invasive behavior of CRC cells. We also validated the prognostic value of the known but understudied molecule IGFBP2 in a secondary CRC dataset. These data indicate that RPPA and other high throughput protein datasets are useful for identifying potential biomarkers and drivers of aggressive tumor behavior, especially for proteins whose RNA expression does not correlate to protein expression, such as GATA3.

Gene expression signature discovery has been dominated by transcript profiling technologies. Since we previously found that a small RPPA dataset from human tumors can be useful as a biological discovery tool ^6^, we tested its utility in a larger dataset from TCGA in this study. In addition to identifying proteins known to drive CRC progression, we identified several novel or understudied proteins associated with recurrence or death of CRC patients. These included IGFBP2 and GATA3, which were identified by multiple statistical methods, and a number of additional proteins that were detected by multiple ( Table 3, Table 4) or any method ( Supplemental Table 3, Supplemental Table 4). Validation of IGFBP2 by TMA staining and GATA3 *in vitro* suggests that our bioinformatic approach has utility and biological validity. Moreover, our analysis showed that GATA3 mRNA levels were not predictive of GATA3 protein levels ( Supplemental Figure 4). Consistent with recent reports showing that RNA and protein expression levels frequently do not correlate with each other ^2, 55^, these data highlight the necessity of incorporating proteomics data into gene signature studies.

Our approach uses a comparison of tumor tissue between good and poor prognosis patients, which differs from previous proteomics studies that have either focused on differences between tumor and normal control tissues or on stage-specific differences ^56– 63^. These studies have given insight in to the pathophysiology of CRC progression. However, our goal was to identify markers that are independent of stage and could be potentially used in the future to predict prognosis in early stage patients. It is agreed that Stage III and IV patients universally benefit from chemotherapy ^64^, but the treatment decision for early Stage II patients is more complicated: there is disagreement over whether Stage II patients should ^65– 67^ or should not ^68, 69^ receive additional chemotherapy. While our findings are clearly a long way away from translation to the clinic, we posit that our general approach has the potential to identify biomarkers that can be used to identify early stage patients that could benefit from additional adjuvant therapy.

A limitation of our study was that the TCGA CRC patient sample set is smaller for RPPA than for more standard analyses such as RNA Seq or DNA mutations (196, compared to 244 and 224 patient samples) ^7^. In addition, many samples either did not have clinical follow-up or had only short follow-up time, further reducing our sample size. Additionally, there were no other published RPPA datasets in CRC that contained analysis of our proteins of interest. Therefore, validation of our findings required either staining of tissue microarrays or *in vitro* experiments. As RPPA datasets accumulate, we anticipate that there will be larger and multiple independent validation datasets with longer follow-up times. Finally, because RPPA is an antibody-based technique, it is usually typically limited in the number of proteins detected. Higher throughput proteomic approaches may solve this problem, although they are often unsuitable for quantitation of posttranslational modifications such as phosphorylation.

We identified increased expression of IGFBP2 to be associated with CRC recurrence and death. High levels of IGFBP2 have been associated with poor prognosis in several cancer types. In breast cancer, IGFBP2 has increased expression compared to normal samples ^70^. IGFBP2 has also been shown to promote invasion of ovarian cancer cells ^71^. In CRC, IGFBP2 has been reported to be upregulated compared to normal colon epithelia ^26^ with a trend towards higher expression in more advanced CRC ^27^. Interestingly, IGFBP2 is expressed predominantly in the crypts of normal colon tissue ( Figure 3a and 28), opposite to the pattern we observed with GATA3 expression and suggesting a stem-cell-like expression pattern. Notably, IGFBP2 has been connected to both hematopoietic and glioma stem cell expansion and survival ^72, 73^. In addition, IGFBP2 overexpression in CRC cell lines was recently found to promote CRC tumorigenesis and metastasis ^28^. Those data are consistent with our finding that high IGFBP2 expression in CRC tumors is significantly associated with death and recurrence in two independent datasets of CRC patients ( Table 3, Table 4; Figure 3).

The top hit in our survival analysis was GATA3, which has not previously been studied in CRC. GATA3 is a transcription factor that was originally identified in T-cells, and controls the differentiation of TH2 cells ^34, 46– 48^, skin cells ^44^, hair follicles ^45^ and luminal cells in the mammary gland ^22, 24^. The importance of GATA3 for mammary luminal cell proliferation and differentiation is suggested by the high expression of GATA3 in luminal breast cancers and recurrent mutations in the luminal subtype that stabilize GATA3 protein expression levels ^23, 25^. Conversely, similar to our findings in CRC, low GATA3 levels are associated with poor patient prognosis in breast cancer ^36– 40^. At this point it is unclear whether that represents the overall poor outcome of non-luminal breast cancers or an active role for GATA3 in suppressing aggressive behavior. Support for the latter possibility is provided by data indicating that re-expression of GATA3 in non-luminal breast cancer cells is sufficient to induce differentiation and suppress lung metastases ^24^.

In CRC, the mechanistic role of GATA3 still remains to be defined. One possibility is that GATA3 controls CRC differentiation, similar to its function in T-cells and luminal breast cells. Consistent with our prediction, IHC stains of normal colon tissue showed higher staining in the superficial mucosa, where the most differentiated cells should be. In addition, the most differentiated CRC cell lines in our panel had the highest GATA3 expression. Additionally, we previously identified three transcriptional subtypes of CRC and then identified subtype-specific driver networks by integrating mutation and copy number alteration data from each subtype with a protein signaling network using a random walk approach ^5^. GATA3 was included in the driver network for the “differentiated subtype” with relatively good survival outcome, although GATA3 mRNA was not significantly up-regulated in this subtype. Another nonexclusive possibility is that GATA3 regulates TGF-β signaling, a key pathway regulating CRC aggressiveness, as reported in breast cancer ^74^. Further work is required to determine if any of these or other mechanisms are responsible for the role of GATA3 in CRC.

## Data availability

F1000Research: Dataset 1. Raw data of identified protein expression and signaling changes statistically associated with patient outcome, 10.5256/f1000research.6388.d46074 ^75^

## Figures and Tables

**Supplemental Table 1.** Hazard ratios and p-values for cox regression analysis identifying proteins associated with death. Only proteins with a significant p-value (<0.05) were included.

*Death*			
	Antibody	Hazard_ Ratio	P_value
All stages	XBP1.G.C	7.12	0.003
	AMPK_alpha.R.C	4.82	0.011
	Tau.M.C	0.19	0.015
	Smad3.R.V	3.48	0.019
	Bid.R.C	0.06	0.028
	INPP4B.G.C	1.96	0.029
	IGFBP2.R.V	1.44	0.030
	GATA3.M.V	0.24	0.038
	Bim.R.V	2.67	0.044
	Fibronectin.R.C	0.58	0.046
Stages I-II	GATA3.M.V	0.04	0.0003
	K.Ras.M.C	0.14	0.002
	cIAP.R.V	15.84	0.005
	IGFBP2.R.V	2.25	0.006
	Rb.M.V	0.02	0.008
	Cyclin_D1.R.V	0.00	0.010
	AMPK_alpha.R.C	24.32	0.012
	X14.3.3_epsilon.M.C	0.00	0.017
	Tau.M.C	0.05	0.025
	Notch3.R.C	3.33	0.036
	PR.R.V	0.02	0.047
Stages I-III	GATA3.M.V	0.07	0.001
	Rb.M.V	0.06	0.004
	Bid.R.C	0.01	0.006
	IGFBP2.R.V	1.83	0.007
	K.Ras.M.C	0.23	0.008
	Tau.M.C	0.07	0.009
	AMPK_alpha.R.C	5.91	0.015
	c.Kit.R.V	2.25	0.027
	TAZ_pS89.R.C	0.00	0.031
	PR.R.V	0.06	0.046
	XBP1.G.C	6.28	0.046

**Supplemental Table 2.** Hazard ratios and p-values for cox regression analysis identifying proteins associated with recurrence. Only proteins with a significant p-value (<0.05) were included.

*Recurrence*			
	Antibody	Hazard_ Ratio	P_value
All stages	COX.2.R.C	2.02	0.001
	YAP_pS127.R.C	2.31	0.021
	Caspase.7_ cleavedD198.R.C	0.63	0.028
	c.Jun_pS73.R.C	4.18	0.042
	MEK1_pS217_S221. R.V	2.42	0.049
Stages I-II	XIAP.R.C	41.60	0.012
	HSP70.R.C	2.44	0.031
	YAP_pS127.R.C	8.12	0.035
	IGFBP2.R.V	2.01	0.040
	COX.2.R.C	2.68	0.045
	PEA.15.R.V	52.19	0.046
	CDK1.R.V	0.00	0.050
Stages I-III	IGFBP2.R.V	1.96	0.004
	COX.2.R.C	2.30	0.007
	YB.1_pS102.R.V	7.69	0.019
	XRCC1.R.C	16.08	0.028
	XIAP.R.C	12.56	0.030
	DJ.1.R.C	0.07	0.043

**Supplemental Table 3.** All proteins associated with death, sorted in descending order by the number of times identified.

*Death*											
	Method	Cox			Wilma			Regsubsets			
	*Stages*	*All*	*I-II*	*I-III*	*All*	*I-II*	*I-III*	*All*	*I-II*	*I-III*	Total
**Antibody**	GATA3.M.V	✔	✔	✔	✔	✔	✔		✔	✔	8
	Bid.R.C	✔		✔		✔	✔		✔	✔	6
	Rb.M.V		✔	✔		✔	✔			✔	5
	AMPK_alpha.R.C	✔	✔	✔	✔	✔					5
	Tau.M.C	✔	✔	✔	✔			✔			5
	IGFBP2.R.V	✔	✔	✔			✔				4
	Beclin.G.V				✔		✔	✔		✔	4
	Src_pY527.R.V				✔	✔			✔	✔	4
	c.Jun_pS73.R.C					✔	✔			✔	3
	X4E.BP1.R.V					✔	✔		✔		3
	Bim.R.V	✔			✔			✔			3
	COX.2.R.C				✔					✔	2
	Smad4.M.V				✔		✔				2
	ERK2.R.NA				✔				✔		2
	PR.R.V		✔	✔							2
	Annexin_I.R.V							✔	✔		2
	Chk1.R.C					✔		✔			2
	K.Ras.M.C		✔	✔							2
	MSH2.M.C				✔				✔		2
	p27_pT157.R.C				✔		✔				2
	p70S6K_pT389.R.V					✔	✔				2
	Smad3.R.V	✔				✔					2
	X4E.BP1_pT37.R.V				✔	✔					2
	XBP1.G.C	✔		✔							2
	ACC1.R.C				✔						1
	Akt.R.V				✔						1
	AMPK_pT172.R.V				✔						1
	Caspase.3_active.R.C						✔				1
	CDK1.R.V				✔						1
	Fibronectin.R.C	✔									1
	STAT5.alpha.R.V					✔					1
	TAZ.R.C					✔					1
	YB.1_pS102.R.V						✔				1
	B.Raf.M.NA				✔						1
	Bax.R.V							✔			1
	c.Kit.R.V			✔							1
	cIAP.R.V		✔								1
	Cyclin_D1.R.V		✔								1
	Cyclin_E1.M.V				✔						1
	eIF4E.R.V							✔			1
	INPP4B.G.C	✔									1
	Notch3.R.C		✔								1
	p27.R.V					✔					1
	p38_pT180_Y182.R.V									✔	1
	PRAS40_pT246.R.V							✔			1
	S6.R.NA							✔			1
	TAZ_pS89.R.C			✔							1
	X14.3.3_epsilon.M.C		✔								1

**Supplementary Table 4.** All proteins associated with recurrence, sorted in descending order by the number of times identified.

*Recurrence*											
	*Method*	Cox			Wilma			Regsubsets			
	*Stages*	*All*	*I-II*	*I-III*	*All*	*I-II*	*I-III*	*All*	*I-II*	*I-III*	Total
**Antibody**	**COX.2.R.C**	✔	✔	✔	✔		✔	✔	✔		7
	**c.Jun_pS73.R.C**	✔			✔		✔	✔		✔	5
	**Rb.M.V**					✔	✔		✔	✔	4
	**IGFBP2.R.V**		✔	✔			✔				3
	**Rb_pS807_S811.R.V**					✔		✔	✔		3
	**Beclin.G.V**				✔		✔				2
	**Smad4.M.V**						✔			✔	2
	GSK3.alpha.beta.M.V				✔		✔				2
	**HSP70.R.C**		✔		✔						2
	**p70S6K.R.V**				✔			✔			2
	**PEA.15.R.V**		✔						✔		2
	PI3K.p85.R.V					✔	✔				2
	XIAP.R.C		✔	✔							2
	**XRCC1.R.C**			✔			✔				2
	YAP_pS127.R.C	✔	✔								2
	GATA3.M.V				✔						1
	ERK2.R.NA							✔			1
	PR.R.V						✔				1
	ACC1.R.C								✔		1
	AMPK_pT172.R.V					✔					1
	Caspase.3_active.R.C				✔						1
	CDK1.R.V		✔								1
	Fibronectin.R.C					✔					1
	STAT5.alpha.R.V							✔			1
	TAZ.R.C				✔						1
	YB.1_pS102.R.V			✔							1
	ACC_pS79.R.V					✔					1
	Akt_pS473.R.V				✔						1
	ARID1A.M.V				✔						1
	Caspase.7_cleavedD198.R.C	✔									1
	DJ.1.R.C			✔							1
	GSK3_pS9.R.V						✔				1
	Lck.R.V					✔					1
	MEK1_pS217_S221.R.V	✔									1
	NF2.R.C					✔					1
	Rad50.M.C					✔					1
	Rad51.M.C									✔	1
	Shc_pY317.R.NA						✔				1
	STAT3_pY705.R.V							✔			1

**Supplemental Table 5.** Characteristics of patients included in TMA analysis.

*TMA Patients*		
*Characteristics*	Average age (years)	63.05
	Male	26
	Female	34
	Gender unknown	1
	Average follow up time (days)	2074.08
*Recurrence*	Average days to recurrence	674.95
	Recurrent	22
	Non-recurrent	39
*Death*	Average days to death	1180.05
	Deceased	21
	Living	40
*Stage*	Stage I	0
	Stage II	34
	Stage III	26
	Stage IV	0
