# Supplementary material for: Linking patient outcome to high throughput protein expression data identifies novel regulators of colorectal adenocarcinoma aggressiveness
Source: F1000Res. 2015 Apr 24;4:99. [Version 1] doi: 10.12688/f1000research.6388.1 (PMC4457132; doi:10.12688/f1000research.6388.1)
Supplement: Peer review history

# Peer review: Referee response for version 1

**DOI:** 10.5256/f1000research.6853.r8886

**Published:** 2015-06-04

**Authors:** Simon Saule

**Affiliations:** 1 Institut Curie, Paris, France

The paper of French *et al.*  "Linking patient outcome to high throughput protein expression data identifies novel regulators of colorectal adenocarcinoma" utilizes publicly available RPPA data for colorectal adenocarcinoma with the objective to predict patient survival. Two factors were identified to be significantly associated with bad prognosis: high levels of IGFBP2 and low levels of GATA binding protein 3.  GATA3 protein level is not correlated with its RNA level, highlighting the interest of RPPA use. a Proteins accumulation in tissue microarray was performed, and biological validation in cell culture was provided for GATA3, through retroviral delivery in colon cancer cell lines. Over expression of GATA3 specifically reduced invasion in a transwell filter assay, and reduced the size of colony formation in matrigel without effect on cell proliferation in 2D cultures. The data are sound, convincing and of interest for the community.

I have read this submission. I believe that I have an appropriate level of expertise to confirm that it is of an acceptable scientific standard.

# Peer review: Referee response for version 1

**DOI:** 10.5256/f1000research.6853.r8773

**Published:** 2015-06-01

**Authors:** Stanley Stylli

**Affiliations:** 1 Department of Surgery, University of Melbourne, Parkville, VIC, Australia

The research article ‘Linking patient outcome to high throughput protein expression data identifies novel regulators of colorectal adenocarcinoma aggressiveness’ by French *et al* presents a solid and well-structured study examining the link between protein signaling data and potentially predicting patient survival, in particular for colorectal cancer. To perform their research, they have utilized publically available databases to detect protein expression changes in colorectal cancer. The appropriateness and robustness of their experimental design is encompassed by the validation through multiple computational methods which identified a number of known, but more importantly new potential regulators of colorectal cancer. Increased levels of IGFBP2 were shown to be associated with tumour recurrence and death. In addition, they also identified that GATA binding protein 3 (GATA3) expression was also associated with patient outcome (being significantly decreased in the lower stage colorectal cancer patients). Confirmation of the role of GATA3 was shown in their proliferation and invasion laboratory studies.  The results are clearly presented and extensively rationalized in the discussion, for which the authors must be commended.  It is a comprehensive study which will be of interest to many readers who wish to undertake similar approaches utilizing protein expression data in public databases as a foundation of their laboratory research. It is an excellent addition to the current literature.

I have read this submission. I believe that I have an appropriate level of expertise to confirm that it is of an acceptable scientific standard.
